# Association between Visual, Hearing and Dual Sensory Impairment and the Frailty Syndrome

**DOI:** 10.14336/AD.2025.0342

**Published:** 2026-05-11

**Authors:** Humberto Yévenes-Briones, Francisco Félix Caballero, Aitana Vázquez-Fernández, Pablo Martinez-Amezcua, Teresa T Fung, Esther Lopez-Garcia

**Affiliations:** ^1^Department of Preventive Medicine and Public Health, School of Medicine, Universidad Autónoma de Madrid, Madrid, Spain.; ^2^Department of Epidemiology, Johns Hopkins Bloomberg School of Public Health, Baltimore, MD, USA.; ^3^Cochlear Center for Hearing and Public Health, Johns Hopkins Bloomberg School of Public Health, Baltimore, MD, USA.; ^4^Department of Nutrition, Harvard T.H. Chan School of Public Health, Boston, MA, USA.; ^5^Department of Nutrition, Simmons University, Boston, MA, USA.

**Keywords:** Visual impairment, hearing impairment, frailty syndrome, UK Biobank

## Abstract

Sensory loss has been associated with multiple adverse health conditions. However, the combined effect of visual and hearing impairment on frailty is unknown. The aim of this study was to examine the association between visual, hearing, and dual-sensory impairment and frailty prevalence. This cross-sectional study investigated 105,406 participants aged ≥39 years from the UK Biobank study. Visual acuity was measured with a chart, as the logarithm of the minimum angle of resolution (LogMAR); functional auditory capacity was measured with a digit triplet test, as the speech reception threshold in noise (SRTn). Dual sensory impairment was defined as the presence of both visual impairment (LogMAR > 0.3 units) and hearing impairment (SRTn ≥ −5.5 dB SNR). To define the frailty syndrome, two methods were used, the frailty phenotype and the FRAIL scale. Analyses were conducted using logistic models adjusted for relevant confounders. Among the participants, 54.3% were women, with a mean age of 56.8 years (SD: 8.1, range 39 to 72). The prevalence of frailty was 3.5%, defined with the frailty phenotype, and 3.6%, using the FRAIL scale. For visual impairment, the OR (95% CI) of frailty was 1.51 (1.28-1.79) for the frailty phenotype, and 1.31 (1.10-1.57), for the FRAIL scale. For hearing impairment, in comparison with having normal hearing, the OR (95% CI) associated with insufficient and poor hearing were 1.32 (1.20-1.45) and 1.83 (1.53-2.19), respectively for the frailty phenotype, and 1.32 (1.19-1.46) and 1.93 (1.60-2.33) for the FRAIL scale. Estimates for the association between dual-sensory impairment and frailty were 2.22 (1.65-2.99) for the frailty phenotype, and 1.73 (1.23-2.42) for the FRAIL scale. Visual and hearing impairments were related to frailty. Having dual-sensory impairment was associated with twice the likelihood of frailty syndrome in comparison with having none of them.

## INTRODUCTION

Frailty is a geriatric syndrome characterized by a decrease in the functioning of multiple physiological systems, accompanied by increased vulnerability to stressors [1]. Frailty is a predictor of poor health outcomes, including longer hospitalization time and greater degree of dependency and mortality [2, 3] with a high burden of social costs, both at the individual level and for families and caregivers [4]. It is an emerging global burden for the health systems and society [5]. Furthermore, many interventions to overcome frailty such as comprehensive geriatric assessment, physical activity or nutritional supplementation have been proven effective in clinical trials but have not consistently shown similar efficacy in routine care [6], so prevention is a fundamental first step.

Over the last 10 years, a great effort has been made to explain the mechanisms of the frailty syndrome [7, 8]. However, how frailty is related to sensory function is little known [9-11]. Sensory impairments increase with age, with visual impairment and hearing impairment being the most prevalent [12]. Both unfavorable conditions are associated with an increased risk of mortality, which is higher when they occur simultaneously [13]. Sensory impairments have also been related to functional limitations [14-17], as well as with multimorbidity [18] and cognitive impairment [19]. Furthermore, both visual and hearing impairment decrease social participation [20, 21], which may reduce leisure-time physical activity and adherence to healthy dietary patterns [22-24], so it seems plausible that these sensory impairments trigger the cascade of events that produce the frailty syndrome [1]. Furthermore, sensory impairments and frailty likely share common biological mechanisms such as chronic inflammation, a hallmark of aging [25-27]. The objective of this study was to examine the association between visual, hearing, and dual-sensory impairment and the frailty syndrome among middle-aged and older adults, to provide a better understanding of the impact of sensory impairments on health.

## METHODS

### Study design and participants

The UK Biobank study is a large population-based cohort study established between 2006 and 2010 in the United Kingdom [28] The study recruited more than 500,000 participants aged 39 to 72 years, with information on their health status, demographics, and lifestyle. In addition, participants provided several types of biological samples and underwent a physical examination. This study was performed under generic ethical approval obtained by the UK Biobank from the NHS National Research Ethics Service (reference 11/NW/0382, June 17, 2011). All study participants signed a written informed consent.

### Definition of sensory impairments

The visual acuity of participants was measured as the logarithm of the minimum angle of resolution (LogMAR) [29]. The UK Biobank system was based on a traditional LogMAR chart: the participant sat on a chair 4 meters from a screen. If the space was limited or if the participant was in a wheelchair, the distance to the screen was set to 3.5 meters. If the participant used glasses for myopia control, they were kept for this test. Of note, it was checked that glasses were clean (offering tissue/wipes if required). If the participant used contact lenses, their use was checked. Then, the left eye was occluded, asking the participant to keep the occluded eye open, if possible. The participant was asked to read out the smallest letter that they could identify on the screen. If they failed, the participant was asked for a larger letter. If the participant could not see even the largest letter, the ‘none’ option was recorded. Next, 5 letters were shown on the display screen. The participant read out each letter starting from the left side of the screen; the test terminated when 2 incorrect values were given. Then, the exam was done with the other eye. Visual impairment was defined as a visual acuity worse than LogMAR 0.3, based on the performance of the 'better-seeing eye,' which is the eye with the greatest visual acuity for each participant [30].

Functional auditory capacity was measured with a digit triplet test (DTT) to determine their speech reception threshold in noise (SRTn), which quantifies the ability to understand speech in noise [31], It was carried out at the UK Biobank evaluation center with the same headphones (Sennheiser HD-25) for all participants. Although the headphones lacked sound-treated booths, the staff kept ambient noise to a minimum to reduce the possibility of distraction by participants. Before starting the test, participants were asked to remove their hearing aids if they had them. In addition, the volume of the speech was set to the individual's most comfortable level for each ear. Then, the participant listened to 15 sets of 3 digits presented with background noise and had to enter each triplet on a keyboard on the touch screen. If the triplet was correctly identified, the noise level would increase for the next triplet; otherwise, the noise level was decreased. Each ear was tested separately, and SRTn was defined as the signal-to-noise ratio (SNR) at which half of the presented digits could be recognized correctly. The SNR could range between −12 and +8 dB. In our analysis, we used the SRTn for the better ear of each participant, that is, the ear with the best hearing performance in the test. If the SRTn was available for only one ear, we assumed it was the better ear. Three cutoff points were established to categorize participants according to their performance on the DTT: (1) normal (SRTn < −5.5 dB SNR); (2) insufficient (SRTn ≥ −5.5 to ≤ −3.5 dB SNR); and (3) poor functional auditory capacity (SRTn > −3.5 dB SNR). The DTT has shown a good correlation with pure-tone audiometry (r = 0.77), which suggests that about 60% of the performance on DTT is explained by standardized audiometric data [32]. We defined dual-sensory impairment when participants had visual impairment and their performance on the DTT was insufficient or poor.

### Frailty

To define the frailty syndrome, two methods were used, the frailty phenotype [1] and the FRAIL scale [33]. The frailty phenotype conceptualizes frailty as a clinical syndrome resulting from altered metabolism coupled with abnormal responses to stress [6, 34]. The frailty phenotype is distinct from the presence of multiple coexisting disorders and disability. The FRAIL scale is an index for frailty screening that differs mainly from the frailty phenotype in the integration of comorbidities, therefore, it approaches the concept of frailty as an accumulation of deficits [34]. In this study, the correlation between both indices was moderate (r=0.65).

For the frailty phenotype, 5 components were evaluated according to the criteria: (1) weight loss, assessed with the question “Compared with one year ago, has your weight changed?”; (2) exhaustion, assessed with the question “Over the past two weeks, how often have you felt tired or had little energy?”; (3) low physical activity, measured using physical activity questionnaire; (4) slowness, assessed with the question “How would you describe your usual walking pace?”; and (5) weakness, measured using grip strength, stratified by sex and body mass index (BMI). For the FRAIL scale the following components were evaluated: (1) fatigue, assessed with the question “Over the past two weeks, how often have you felt tired or had little energy?”; (2) low strength, assessed with the question “Do you get a pain in either leg on walking? ”, (3) reduced aerobic capacity, assessed with the question “Do you get short of breath walking with people of your own age on level ground?”; (4) having several chronic illnesses, based on self-reported diseases; and (5) significant unintentional weight loss, assessed with the question “Compared with one year ago, has your weight changed?”. The frailty syndrome was defined as having ≥3 criteria for both frailty phenotype and FRAIL scale. The details of each component are explained in Supplementary Table 1.

### Potential confounding variables

Participants reported their age, sex, ethnicity, and educational level. The Townsend Deprivation Index (TDI) was calculated based on previous national census outcome areas [35]. Tobacco smoking was self-reported. Weight and height were measured under standardized conditions, and the BMI was calculated as weight (kilograms) divided by height (meters) squared. To determine the hours of sedentary time, we summed up the hours that participants spent watching television, using the computer, and driving on a typical day (ranging between 0 and 16 hours), as in a previous study [36]. Information on the number of hours of sleep per day and number of treatments/medications taken was self-reported.

Diet information was assessed at baseline with up to five 24-hour recalls (Oxford Web-Q)[37] to obtain the average food consumption reflecting habitual diet, and then, nutrient intakes were derived from food composition tables specific to the United Kingdom [38] Among participants who completed at least one dietary 24-hour recalls, we calculated the energy intake, alcohol consumption and the Dietary Approaches to Stop Hypertension (DASH) score, to define diet quality [39]. Physical activity (in metabolic equivalent tasks, METs-h/week) was evaluated with the Short International Physical Activity Questionnaire [40].

The information on falls was obtained from the following question: “In the last year have you had any falls?” The possible answers were “no falls”, “only one fall”, and “more than one fall”. His variable was also categorized into the following categories: no falls, only one fall, or more than one fall [41]. Cognitive function was assessed using a reaction time test; longer reaction times indicated poorer cognitive function [42]. We defined poor cognitive performance as a reaction time greater than or equal to the 75th percentile of the cohort. The diagnosis of depression was self-reported by the participants. Finally, the following three UK Biobank factors were used to define social support: living with a spouse or partner, participation in social activities, and confiding in others. The degree of social support was classified as low (0–1 factor), medium (2 factors), or high (3 factors). Data on social support were available for only 101,786 participants [43].

### Statistical analysis

Of the 111,255 participants with valid visual acuity and functional auditory capacity data, we excluded those participants without data on the frailty phenotype or FRAIL scale variables (n=4836). We also excluded 1013 participants who did not have data on the potential confounding variables. Therefore, statistical analyses were conducted with 105,406 participants.

In this cross-sectional analysis, we assessed differences in sociodemographic characteristics, lifestyle, and number of treatments/medications between sensory impairment status, by means of the analysis of variance and χ2 test, for continuous and categorical variables, respectively. Then, we used logistic regression models to estimate odds ratios (ORs) and 95% confidence intervals (CIs) for the association between each sensory impairment and frailty prevalence.

We built 3 sequentially adjusted models to observe the effect of each of the main confounders on the studied associations. The first model was adjusted for age and sex. A second model was additionally adjusted for ethnic background (British, other ethnic group), educational level (primary or less, secondary, and university), Townsend deprivation index (continuous), tobacco smoking status (current, former, or never), BMI (< 25.0, 25.0–29.9, ≥ 30.0 kg/m^2^), sedentary time (tertiles of h/day), and hours of daily sleep (<7, 7-8, >8). Subsequently, we built a third model additionally adjusted for number of treatments/medications to understand how the effects of poorer health had an impact on the prevalence of frailty. Then, we replicated the main analyses for dual-sensory impairment.

We ran models adjusted for diet quality and physical activity to understand the effect of diet and physical activity on the association between sensory impairments and frailty. This analysis was included as a sensitivity result due to the high number of missing values on these variables (diet quality, n missing= 35,415; physical activity, n missing=18,204). Thus, missing imputation was performed; this was done through multiple imputations by chained equations. Additionally, to understand the effect of critical variables on the relationship between sensory impairment and frailty syndrome, we performed sensitivity analyses, adjusting the models for falls, cognitive performance, depression, and social support.

Next, we performed a joint analysis to assess whether the combined associations of visual impairment and hearing impairment varied with respect to the prevalence of frailty. The reference category represented neither visual nor hearing impairment. Then, to determine whether there were differences between participants who used corrective glasses or hearing aids, we examined the association between categories of hearing and visual function and frailty syndrome, as well as the association between sensory impairment and frailty syndrome.

We also performed stratified analyses by age, sex, Townsend deprivation index, BMI and sedentary time. Lastly, we examined the association between sensory impairments and individual components of the frailty syndrome. All tests were 2-sided, with *P* less than 0.05 considered to be statistically significant. Analyses were performed with Stata (version 17.0; Stata Corp).

**Table 1.** Baseline participants’ characteristics according to sensory impairment status in the UK Biobank. (N=105,406).

	Sensory impairment				
	Without impairment	Visual	Hearing	Dual	*P* value
**N**	90,598	2271	12,033	504	
**%**	85.9	2.2	11.4	0.5	
**Women, %**	54.5	55.0	53.1	54.8	<0.001
**Age, y**	56.3 (8.1)	58.2 (7.6)	60.2 (7.4)	61.2 (6.6)	0.04
**British ethnicity, %**	85.0	80.7	71.2	61.5	<0.001
**Educational level; primary or less, %**	12.9	20.7	25.5	34.3	<0.001
**Townsend deprivation index**	-1.16 (2.9)	-0.58 (3.1)	-0.45 (3.2)	0.22 (3.4)	<0.001
**Current smoker, %**	9.6	10.9	10.3	13.9	<0.001
**Body mass index, kg/m^2^**	27.3 (4.7)	27.5 (4.9)	27.8 (4.9)	27.7 (5.0)	<0.001
**Sedentary time^1^, h/d**	4.9 (2.4)	4.7 (2.5)	4.9 (2.6)	4.6 (2.7)	<0.001
**Hours of daily sleep**	7.1 (1.2)	7.0 (1.4)	7.1 (1.5)	6.9 (1.9)	<0.001
**Nº of treatments/medications taken, %**	2.1 (2.4)	2.3 (2.6)	2.8 (2.9)	3.5 (3.4)	<0.001
**Energy intake, kcal/day^2^**	2090 (556)	2070 (561)	2070 (558)	2032 (501)	<0.001
**Alcohol intake, g/d^2^**	16.6 (21.9)	15.9 (22.1)	14.3 (21.2)	15.8 (22.7)	<0.001
**DASH score^2^**	23.6 (5.0)	23.3 (5.0)	23.8 (4.9)	22.7 (4.9)	<0.001
**Physical activity, METs-h/wk^2^**	44.4 (44.2)	46.6 (46.6)	46.2 (46.3)	46.0 (48.1)	<0.001
**Frailty phenotype, %**	3.0	5.5	6.5	13.1	<0.001
**FRAIL scale, %**	3.1	4.9	6.2	10.1	<0.001

Values are means (SD) unless otherwise indicated. P values based on analysis of variance test for continuous variables or χ2 test for qualitative variables.

1 Total number of hours spent watching television, using the computer, or driving.

2 Description of variables based on participants with data: for energy and alcohol intake and DASH score, n=69,991; for physical activity, n=87,202.

## RESULTS

Among the 105,406 study participants, 54.3% were women and the mean age was 56.8 years (SD: 8.1, range 39 to 72). The prevalence of frailty was 3.5% (3.1% for men and 3.9% for women) and 3.6% (3.0% for men and 4.0% for women) depending on whether the frailty phenotype or the FRAIL scale was used, respectively. In addition, the prevalence of frailty by age was 3.3% and 3.8% for the frailty phenotype, and between 3.8% and 3.2% for the FRAIL scale, for individuals under 60 years and those aged 60 years or older, respectively.

Participants' characteristics according to sensory impairment status are shown in Table 1. In the analytical sample, 2.2% of participants had visual, 11.4% had hearing and 0.5% had dual-sensory impairment. In comparison with participants without impairment, those with dual-sensory impairment were more frequently women, older, of non-British ethnicity, with lower educational level and more deprived, according to the social deprivation index; also, they were more often current smokers. In addition, they were less sedentary, slept less, and used a greater number of medications.

**Table 2.** Odds ratios (95% confidence interval)^1^ for the association between categories of hearing and visual function and frailty syndrome in the UK Biobank. (N= 105,406)

Frailty phenotype							
Visual Function				Hearing function			
	Normal vision (≤ 0.3 logMAR units)	Vision impairment (> 0.3 logMAR units	Normal (SRTn < -5.5 dB SNR)	Insufficient (SRTn -5.5 to -3.5 dB SNR)	Poor (SRTn > -3.5 dB SNR)	P for trend	Per 2-dB SNR increment in SRTn
**N**	102,631	2775	92,869	10,846	1691		105,406
**Cases**	3508	190	2856	665	177		3698
**Age- and sex-adjusted**	1.00	2.00 (1.72-2.33)	1.00	1.97 (1.80-2.15)	3.52 (3.00-4.14)	<0.001	1.36 (1.32-1.40)
**Model 1**	1.00	1.60 (1.37-1.88)	1.00	1.44 (1.31-1.58)	2.15 (1.81-2.55)	<0.001	1.20 (1.17-2.24)
**Model 2**	1.00	1.56 (1.32-1.84)	1.00	1.33 (1.20-1.46)	1.85 (1.53-2.21)	<0.001	1.17 (1.13-1.20)
**Model 3**	1.00	1.51 (1.28-1.79)	1.00	1.32 (1.20-1.45)	1.83 (1.53-2.19)	<0.001	1.16 (1.13-1.20)
**FRAIL scale**							
**N**	102,631	2,775	92,869	10,846	1,691		105,406
**Cases**	3584	162	2950	627	169		3746
**Age- and sex-adjusted**	1.00	1.73 (1.47-2.03)	1.00	1.95 (1.18-2.13)	3.63 (3.08-4.28)	<0.001	1.36 (1.33-1.40)
**Model 1**	1.00	1.41 (1.19-1.67)	1.00	1.45 (1.32-1.60)	2.30 (1.93-2.74)	<0.001	1.23 (1.19-1.26)
**Model 2**	1.00	1.34 (1.12-1.60)	1.00	1.32 (1.20-1.46)	1.94 (1.61-2.34)	<0.001	1.18 (1.15-1.22)
**Model 3**	1.00	1.31 (1.10-1.57)	1.00	1.32 (1.19-1.46)	1.93 (1.60-2.33)	<0.001	1.18 (1.14-1.22)

1 From logistic regression models.

Model 1: additionally adjusted for ethnic background (British, other ethnic group), educational level (primary or less, secondary, and university), Townsend deprivation index (continuous), smoking status (current, former, or never), BMI (< 25.0, 25.0–29.9, ≥ 30.0 kg/m^2^), sedentary time (tertiles of h/day) and hours of daily sleep (<7, 7-8, >8).

Model 2: additionally adjusted for number of treatments/medications.

Model 3: additionally adjusted for visual or hearing impartment, as appropriate.

We observed an association between categories of hearing and visual function and frailty syndrome (Table 2). For visual impairment, the fully-adjusted OR (95% CI) were 1.51 (1.28-1.79) for the frailty phenotype, and 1.31 (1.10-1.57) for the FRAIL scale. For hearing function, in comparison with having normal hearing, the OR of frailty associated with insufficient and poor hearing were 1.32 (1.20-1.45) and 1.83 (1.53-2.19), respectively for the frailty phenotype, and 1.32 (1.19-1.46) and 1.93 (1.60-2.33) for the FRAIL scale. In addition, each 2-dB increment in the SRTn was associated with a 16% (13%-20%) and 18% (14%-22%) increased likelihood of the frailty syndrome, considering both scales.

We compared how having sensory impairments was related to frailty in Table 3. For the frailty phenotype, in comparison with not having sensory impairment, the fully-adjusted OR (95% CI) associated with visual impairment was 1.50 (1.23-1.84), for hearing impairment was 1.39 (1.27-1.52), and for dual-sensory impairment was 2.22 (1.65-2.99). Using the FRAIL scale, the OR associated with visual impairment was 1.36 (1.10-1.68), for hearing impairment was 1.42 (1.29-1.56), and for dual-sensory impairment was 1.73 (1.23-2.42). The results were similar when models were additionally adjusted for diet quality and physical activity (Supplementary Table 2). Furthermore, the results remained similar after additional adjustments for falls, poor cognitive performance, depression, and social support (Supplementary Table 3 and 4).

**Figure 1.**
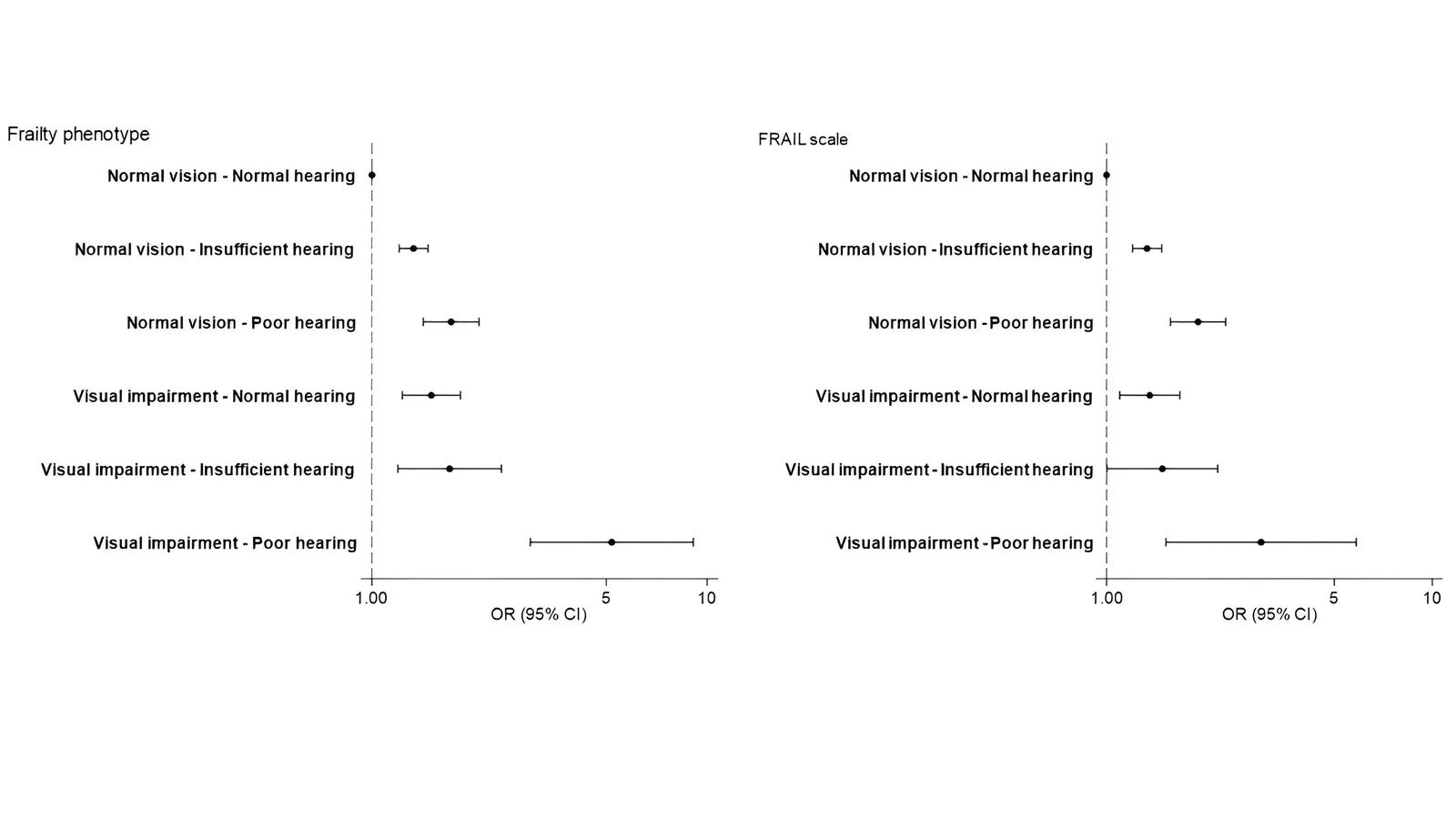
**Odd ratios and 95% CI, from logistic regression models, for the joint analysis between visual and hearing function and frailty**. Models were adjusted for age, sex, ethnic background (British, other ethnic group), educational level (primary or less, secondary, and university), Townsend deprivation index (continuous), smoking status (current, former, or never), BMI (< 25.0, 25.0–29.9, ≥ 30.0 kg/m^2^), sedentary time (tertiles of h/day), hours of daily sleep (<7, 7-8, >8) and number of treatments/medications taken.

Figure 1 showed that those participants who had visual impairment and poor hearing were more likely to have frailty syndrome than any other combination of function. In the sensitivity analysis that included only participants using devices to correct visual and hearing limitations, the results were like the main analyses (Supplementary Table 5 and 6).

The analysis by subgroups of participants showed different associations between hearing impairment and frailty by age and sedentary time subgroups, those younger and less sedentary shower stronger associations. Differences in the association between dual sensory impairment and frailty by BMI levels were also observed but only for one of the definitions of frailty (Supplementary Table 7). Finally, in separate analyses for each criterion of frailty, we observed that the components that had the greatest weight in the association between visual, hearing and dual-sensory impairment and the frailty phenotype was “slowness”; for FRAIL scale was “reduced aerobic capacity” (Supplementary Table 8).

## DISCUSSION

In this cross-sectional analysis, visual, hearing, and dual-sensory impairment were associated with the frailty syndrome independently of sociodemographic and lifestyle characteristics, and number of treatments/medications taken. Dual-sensory impairment was associated with twice the likelihood of frailty, in comparison with having none of them. Although we may hypothesize a causal relation between visual, hearing and dual sensory impairment and frailty, we can also speculate that the association is explained by shared underlying pathological processes or that the sensory impairments examined are only early physiological markers of the frailty syndrome.

**Table 3.** Odds ratios (95% confidence interval)^1^ for the association between sensory impairment and frailty syndrome in the UK Biobank. (N= 105,406).

	Sensory impairment			
	Without any impairment (n=90,598)	Visual (n=2271)	Hearing (n=12,033)	Dual (n=504)
**Frailty phenotype**				
Cases	2,732	124	776	66
Age- and sex-adjusted	1.00	1.81 (1.51-2.18)	2.12 (1.95-2.31)	4.57 (3.51-5.94)
Model 1	1.00	1.50 (1.24-1.81)	1.52 (1.39-1.66)	2.72 (2.06-3.59)
Model 2	1.00	1.50 (1.23-1.84)	1.39 (1.27-1.52)	2.22 (1.65-2.99)
**FRAIL scale**				
Cases	2,839	111	745	51
Age- and sex-adjusted	1.00	1.62 (1.33-1.97)	2.13 (1.96-2.32)	3.67 (2.74-4.92)
Model 1	1.00	1.36 (1.11-1.66)	1.56 (1.43-1.71)	2.27 (1.66-3.09)
Model 2	1.00	1.36 (1.10-1.68)	1.42 (1.29-1.56)	1.73 (1.23-2.42)

1 From logistic regression models.

Model 1: additionally adjusted for ethnic background (British, other ethnic group), educational level (primary or less, secondary, and university), Townsend deprivation index (continuous), smoking status (current, former, or never), BMI (< 25.0, 25.0–29.9, ≥ 30.0 kg/m^2^), sedentary time (tertiles of h/day) and hours of daily sleep (<7, 7-8, >8).

Model 2: additionally adjusted for number of treatments/medications.

These results are consistent with the previous literature. In a cross-sectional study using data from the National Health and Nutrition Examination Survey, Swenor et al. [44] observed an association between visual impairment and the frailty syndrome in a population over 60 years of age. Regarding hearing loss, in a previous study, we observed a strong association between lower values of pure tone average measurements and the frailty syndrome [14]. Only Zhao et al. [10], using the West China Health and Aging Trend survey, had previously assessed the association between dual-sensory impairment and frailty, with self-reported information on visual acuity and hearing function.

Visual and hearing impairments are two adverse health conditions highly prevalent. Globally, it is estimated that more than 2.2 billion people are visually impaired or blind [45] while more than 1.5 billion live with hearing problems [46]. Both systems, the visual and auditory, play an important role in the independence and functioning of individuals, allowing interaction with the physical environment and in social context [47]. Visual impairment may affect people's ability to engage in adequate physical activity [22] and increase the risk of falls [48], restricting social participation [20]. On the other hand, hearing impairment affects people's ability to communicate, increasing loneliness and social isolation [49]. In addition, hearing impairment increases the risk of falls [50]. Finally, both disabilities increase social isolation, which may impair diet quality due to a lack of motivating factors in food preparation in social settings [9, 24]. Besides, the co-occurrence of sensory impairments decreases the intrinsic capacity of the individual, producing loss of capacities, which in turn increases the risk of frailty [51]. All these processes could accelerate the progression of frailty.

We observed that having visual impairment and having a more severe degree of hearing impairment increased the probability of having frailty syndrome compared to not having any disability and was greater than any other combination of sensory function. This adds evidence that the problems faced by people with dual-sensory impairment are considerably greater compared to those with just one impairment [47]. On the other hand, our findings showed a stronger association among those younger than 60 years and we can speculate that the impact of sensory loss is greater when it happens earlier in life. However, it is unclear the interpretation of a stronger association among those reporting less sedentary time, as well as for those with lower BMI.

Regarding the analysis of each of the components of the indices, we observed that “slowness” and “reduced aerobic capacity” were the ones that contributed most to the association. The “slowness” component has been shown to be one of the best predictors of functional decline in adults [52]. On the other hand, the greater effect of the “reduced aerobic capacity” component can be justified because reducing aerobic capacity could reduce physical activity; increasing physical activity has been shown to reduce the risk of frailty [52].

The biology under the frailty syndrome is still under study. Among the most studied mechanisms are cellular senescence, mitochondrial dysfunction, unregulated nutrient detection and chronic inflammation [7, 53, 54]. In this same way, chronic inflammation inhibits growth factor expression and increases catabolism, thus contributing to sarcopenia and frailty [55]. Inflammation, which can occur in response to non-infectious triggers such as cellular senescence and mitochondrial dysfunction, is also responsible for some of the mechanisms present in both visual56 and hearing impairment [57, 58]. Another key mechanism in frailty is mitochondrial dysfunction, which alters mitochondrial homeostasis and results in lower cellular energy, increased production of reactive oxygen species and inflammation; it is also a biological mechanism shared with visual and hearing impairment [6, 26, 59, 60]. In this line, visual and hearing impairment could be physiological markers of frailty, mainly due to shared biological and neurodegenerative processes.

This work has several strengths. Two objective measures were used to assess visual and hearing functions. In addition, two measures of frailty were considered, both widely used in epidemiological studies, although very different in the type of information included. In addition, these analyses were adjusted for major confounders. The main limitation of our study was the cross-sectional design, since cases of incident frailty were insufficient in this cohort to assess the longitudinal association; therefore, we could not attribute directionality to the observed associations. In addition, another limitation is the difference between the two sensory evaluation procedures: glasses were used for the visual test, but no hearing aids were provided for the hearing test. This discrepancy may have led to misclassification of subjects with sensory impairments. Finally, in the UK Biobank population, there is evidence of selection bias toward 'healthy volunteers,' as it is not a representative sample of the general UK population; therefore, generalizations should be made with caution.

In conclusion, vision, hearing and especially dual-sensory impairment were associated with frailty among middle-aged and older people. Having dual-sensory impairment was associated with twice the likelihood of frailty syndrome in comparison with having none of them. The findings of this research could help detect people at high risk of frailty. Furthermore, these results may encourage the evaluation of frailty status among those who have visual and hearing impairment. Additional well-designed longitudinal studies with long-term follow-ups are needed to provide more evidence supporting the association between visual, hearing and dual-sensory impairment and the risk of the frailty syndrome.

## Supplementary Materials

The Supplementary data can be found online at: www.aginganddisease.org/EN/10.14336/AD.2025.0342.
