# Supplementary material for: Association between Visual, Hearing and Dual Sensory Impairment and the Frailty Syndrome
Source: Aging Dis. 2025 May 11;17(3):1700–9. doi: 10.14336/AD.2025.0342 (PMC13061544; doi:10.14336/AD.2025.0342)
Supplement: Supplementary file 1 — The Supplementary data can be found online at: www.aginganddisease.org/EN/10.14336/AD.2025.0342. [file AD-17-3-1700-s.pdf]

SUPPLEMENTARY DATA

Association between Visual, Hearing and Dual Sensory Impairment and the Frailty Syndrome

Humberto Yévenes-Briones, Francisco Félix Caballero, Aitana Vázquez-Fernández, Pablo Martinez-Amezcua, Teresa T Fung, Esther Lopez-Garcia

© 2025. Yévenes-Briones H et al. Published online at http://www.aginganddisease.org/EN/10.14336/AD.2025.0342

SUPPLEMENTARY DATA

Supplementary Table 1. Definitions of components from the frailty phenotype and the FRAIL scale in the UK Biobank.

**Frailty phenotype**	**Self-reported question/ measurement in UK Biobank**	**Scoring**
Weight loss	"Compared with one year ago, has your weight changed?"	1=Yes, lost weight<br>0=Other
Exhaustion	"Over the past two weeks, how often have you felt tired or had little energy?"	1=More than half the days or nearly every day<br>0=Other
Low physical activity	Physical activity questionnaire, categorized into 4 levels:<br>1. none – no physical activity in the last 4 weeks<br>2. low – light DIY activity (e.g., pruning, watering the lawn) only in the last 4 weeks<br>3. medium – heavy DIY activity (e.g., weeding, lawn mowing, carpentry and<br>digging), walking for pleasure, or other exercises in the last 4 weeks<br>4. high – strenuous sports in the last 4 weeks	1=None, or light activity with a frequency of once per week or less<br>0=Medium or heavy activity, or light activity more than once per week
Slowness	"How would you describe your usual walking pace?"	1=Slow<br>0=Other
Weakness	Measured grip strength (maximum value of either hand), stratified by sex and body mass index (BMI)	1= Cohort-specific lowest quintile of grip strength in dominant hand, adjusted for sex and body mass index<br>0= Other
**FRAIL scale**	**Self-reported question/ measurement in UK Biobank**	**Scoring**
Fatigue	"Over the past two weeks, how often have you felt tired or had little energy?"	1=More than half the days or nearly every day<br>0=Other
Low strength	"Do you get a pain in either leg on walking? "	1=Yes<br>0=No
Reduced aerobic capacity	"Do you get short of breath walking with people of your own age on level ground?"	1= Yes<br>0= No
Having several chronic illnesses	Self-report of diseases was used to cancer, hypertension, type 2 diabetes, angina, myocardial infarction, stroke, congestive heart failure, asthma, chronic obstructive lung disease, arthritis, Parkinson's disease, kidney disease and depression.	1= If the participants reported at least 5 diseases<br>0= Other
Unintentional weight loss	"Compared with one year ago, has your weight changed?"	1=Yes, lost weight<br>0=Other

Supplementary Table 2. Odds ratios (95% confidence interval)[1] for the association between sensory impairment and frailty syndrome in the UK Biobank, with additional adjustment for total energy intake, alcohol consumption, diet quality, and physical activity. (N= 105,406)

	**Sensory impairment**			
	**Without any impairment (n=90,598)**	**Visual (n=2271)**	**Hearing (n=12,033)**	**Dual (n=504)**
Frailty phenotype				
Cases	2,732	124	776	66
Age- and sex-adjusted	1.00	1.81 (1.51-2.18)	2.12 (1.95-2.31)	4.57 (3.51-5.94)
Model 1	1.00	149 (1.23-1.81)	1.50 (1.38-1.64)	2.57 (1.93-3.42)
Model 2	1.00	1.49 (1.22-1.81)	1.48 (1.22-1.81)	2.44 (1.83-3.26)
FRAIL scale				
Cases	2,839	111	745	51
Age- and sex-adjusted	1.00	1.62 (1.33-1.97)	2.13 (1.96-2.32)	3.67 (2.74-4.92)
Model 1	1.00	1.35 (1.10-1.65)	1.55 (1.41-1.69)	2.17 (1.58-2.97)
Model 2	1.00	1.33 (1.08-1.63)	1.51 (1.38-1.66)	2.03 (1.48-2.79)

Abbreviations: VI Visual impairment, HI Hearing impairment, DSI Dual-sensory impairment
[1]From logistic regression models.
Model 1: additionally adjusted for ethnic background (British, other ethnic group), educational level (primary or less, secondary, and university), Townsend deprivation index (continuous), smoking status (current, former, or never), BMI (< 25.0, 25.0–29.9, ≥ 30.0 kg/m^2), sedentary time (tertiles

© 2025. Yévenes-Briones H et al. Published online at http://www.aginganddisease.org/EN/10.14336/AD.2025.0342

of h/day), hours of daily sleep (<7, 7-8, >8), energy intake (tertiles of kcal/d), alcohol consumption (quintiles of g/d), DASH diet (tertiles of score), and physical activity (tertiles of METs).
Model 2: additionally adjusted for number of treatments/medications.

Supplementary Table 3. Odds ratios (95% confidence interval) for the association between sensory impairment and frailty syndrome in the UK Biobank, with additional adjustment for falls during the last year before the interview. (N= 105,406)

	**Sensory impairment**			
	**Without any impairment (n=90,598)**	**Visual (n=2271)**	**Hearing (n=12,033)**	**Dual (n=504)**
Frailty phenotype				
Cases	2,732	124	776	66
Fully adjusted model	1.00	1.47 (1.20-1.79)	1.35 (1.23-1.49)	2.11 (1.56-2.85)
FRAIL scale				
Cases	2,839	111	745	51
Fully adjusted model	1.00	1.31 (1.06-1.63)	1.38 (1.25-1.52)	1.61 (1.14-2.27)

Logistic regression model adjusted for ethnic background (British, other ethnic group), educational level (primary or less, secondary, and university), Townsend deprivation index (continuous), smoking status (current, former, or never), BMI (< 25.0, 25.0–29.9, ≥ 30.0 kg/m^2), sedentary time (tertiles of h/day), hours of daily sleep (<7, 7-8, >8), number of treatments/medications and falls in the last year (no, only one fall or more than one fall).

Supplementary Table 4. Odds ratios (95% confidence interval) for the association between sensory impairment and frailty syndrome in the UK Biobank, with additional adjustment for poor cognitive performance, depression, and social support. (N= 101,786)

	**Sensory impairment**			
	**Without any impairment (n=90,598)**	**Visual (n=2271)**	**Hearing (n=12,033)**	**Dual (n=504)**
Frailty phenotype				
Cases	2611	117	712	59
Fully adjusted model	1.00	1.44 (1.17-1.77)	1.32 (1.20-1.45)	1.92 (1.40-2.64)
FRAIL scale				
Cases	2722	105	697	47
Fully adjusted model	1.00	1.31 (1.05-1.63)	1.39 (1.26-1.54)	1.59 (1.12-2.26)

Logistic regression model adjusted for ethnic background (British, other ethnic group), educational level (primary or less, secondary, and university), Townsend deprivation index (continuous), smoking status (current, former, or never), BMI (< 25.0, 25.0–29.9, ≥ 30.0 kg/m^2), sedentary time (tertiles of h/day), hours of daily sleep (<7, 7-8, >8), number of treatments/medications, poor cognitive performance (no or yes), depression (no or yes) and social support (low, medium or high).

Supplementary Table 5. Odds ratios (95% confidence interval)[1] for the association between categories of hearing and visual function and frailty syndrome among participants using devices to correct visual and hearing limitations in the UK Biobank. (N= 93,596)

	**Frailty phenotype**						
	**Visual Function**		**Hearing function**				
	Normal vision (≤ 0.3 logMAR units)	Vision impairment (> 0.3 logMAR units	Normal (SRTn < -5.5 dB SNR)	Insufficient (SRTn -5.5 to -3.5 dB SNR)	Poor (SRTn > -3.5 dB SNR)	P for trend	Per 2-dB SNR increment in SRTn
N	91,038	2558	82,117	9919	1560		90,191

© 2025. Yévenes-Briones H et al. Published online at http://www.aginganddisease.org/EN/10.14336/AD.2025.0342

Cases	3232	173	2,629	612	164		3405
Age- and sex-adjusted	1.00	1.93 (1.65-2.26)	1.00	1.93 (1.77-2.12)	3.47 (2.93-4.10)	<0.001	1.36 (1.32-1.40)
Model 1	1.00	1.55 (1.31-1.83)	1.00	1.42 (1.29-1.57)	2.15 (1.80-2.57)	<0.001	1.21 (1.17-1.24)
Model 2	1.00	1.50 (1.26-1.78)	1.00	1.30 (1.18-1.44)	1.82 (1.51-2.20)	<0.001	1.17 (1.13-1.21)
Model 3	1.00	1.46 (1.22-1.73)	1.00	1.30 (1.17-1.43)	1.80 (1.49-2.18)	<0.001	1.16 (1.12-1.20)

N	87,806	2407	82,117	9,919	1,560		90,213
Cases	3232	151	2656	573	154		3383
Age- and sex-adjusted	1.00	1.72 (1.46-2.04)	1.00	1.93 (1.76-2.12)	3.55 (2.99-4.22)	<0.001	1.36 (1.33-1.40)
Model 1	1.00	1.40 (1.18-1.67)	1.00	1.44 (1.31-1.59)	2.27 (1.89-2.73)	<0.001	1.23 (1.19-1.26)
Model 2	1.00	1.33 (1.10-1.61)	1.00	1.30 (1.17-1.45)	1.88 (1.54-2.29)	<0.001	1.18 (1.14-1.22)
Model 3	1.00	1.31 (1.08-1.58)	1.00	1.30 (1.17-1.44)	1.87 (1.53-2.27)	<0.001	1.18 (1.14-1.22)

[1]From logistic regression models.
Model 1: additionally adjusted for ethnic background (British, other ethnic group), educational level (primary or less, secondary, and university), Townsend deprivation index (continuous), smoking status (current, former, or never), BMI (< 25.0, 25.0–29.9, ≥ 30.0 kg/m^2), sedentary time (tertiles of h/day) and hours of daily sleep (<7, 7-8, >8).
Model 2: additionally adjusted for number of treatments/medications.
Model 3: additionally adjusted for visual or hearing impartment, as appropriate.

Supplementary Table 6. Odds ratios (95% confidence interval)[1] for the association between sensory impairment and frailty syndrome among participants using devices to correct visual and hearing limitations in the UK Biobank. (N= 93,596)

	**Sensory impairment**			
	**Without any impairment (n=90,598)**	**Visual (n=2271)**	**Hearing (n=12,033)**	**Dual (n=504)**
Frailty phenotype				
Cases	2515	114	717	59
Age- and sex-adjusted	1.00	1.76 (1.45-2.14)	2.09 (1.92-2.28)	4.26 (3.23-5.62)
Model 1	1.00	1.45 (1.19-1.77)	1.50 (1.37-1.65)	2.60 (1.94-3.48)
Model 2	1.00	1.44 (1.17-1.78)	1.37 (1.24-1.50)	2.11 (1.54-2.89)
FRAIL scale				
Cases	2550	106	682	45
Age- and sex-adjusted	1.00	1.66 (1.36-2.02)	2.12 (1.94-2.32)	3.41 (2.50-4.65)
Model 1	1.00	1.38 (1.12-1.70)	1.55 (1.41-1.70)	2.14 (1.54-2.98)
Model 2	1.00	1.37 (1.10-1.71)	1.39 (1.26-1.54)	1.62 (1.13-2.31)

[1]From logistic regression models.
Model 1: additionally adjusted for ethnic background (British, other ethnic group), educational level (primary or less, secondary, and university), Townsend deprivation index (continuous), smoking status (current, former, or never), BMI (< 25.0, 25.0–29.9, ≥ 30.0 kg/m^2), sedentary time (tertiles of h/day) and hours of daily sleep (<7, 7-8, >8).
Model 2: additionally adjusted for number of treatments/medications.

© 2025. Yévenes-Briones H et al. Published online at http://www.aginganddisease.org/EN/10.14336/AD.2025.0342

Supplementary Table 7. Odds ratios (95% confidence interval) for the association between categories of hearing and visual function and frailty syndrome, by subgroups of participants in the UK Biobank. (N= 105,406)

	Sensory impairment			
	**Without any impairment**	**Visual**	**Hearing**	**Dual**
Frailty phenotype				
Age				
< 60 y, n= 57,575	1.00	1.63 (1.23-2.18)	1.61 (1.40-1.86)*	2.70 (1.65-4.41)
≥ 60 y, n= 47,831	1.00	1.42 (1.08-1.87)	1.28 (1.14-1.45)*	2.02 (1.39-2.93)
Sex				
Men, n=48,137	1.00	1.46 (1.07-2.01)	1.38 (1.20-1.59)	2.04 (1.29-3.23)
Women, n=57,269	1.00	1.51 (1.17-1.96)	1.39 (1.23-1.57)	2.31 (1.56-3.43)
Townsend deprivation index				
≤ median, n=52,710	1.00	1.20 (0.82-1.79)	1.38 (1.17-1.64)	1.25 (0.56-2.78)
>median, n=52,696	1.00	1.69 (1.35-2.13)	1.44 (1.29-1.61)	2.69 (1.95-3.72)
BMI, kg/m^2				
<25, n= 35,583	1.00	1.54 (1.01-2.34)	1.46 (1.19-1.78)	2.83 (1.56-5.13)
≥25, n= 69,823	1.00	1.50 (1.20-1.88)	1.38 (1.25-1.53)	2.10 (1.50-2.94)
Sedentary time, h/day				
≤ median, n=56,191	1.00	1.77 (1.34-2.34)	1.57 (1.36-1.80)*	2.36 (1.56-3.57)
>median, n=49,215	1.00	1.29 (0.97-1.72)	1.27 (1.12-1.44)*	2.03 (1.32-3.13)
FRAIL scale				
Age				
< 60 y, n= 57,575	1.00	1.42 (1.06-1.89)	1.65 (1.44-1.89)*	2.41 (1.45-3.99)
≥ 60 y, n= 47,831	1.00	1.26 (0.92-1.72)	1.20 (1.05-1.37)*	1.23 (0.77-1.95)
Sex				
Men, n=48,137	1.00	1.38 (0.99-1.93)	1.34 (1.15-1.55)	1.65 (0.98-2.77)
Women, n=57,269	1.00	1.32 (1.00-1.73)	1.45 (1.29-1.64)	1.75 (1.12-2.72)
Townsend deprivation index				
≤ median, n=52,710	1.00	1.27 (0.85-1.90)	1.38 (1.15-1.64)	1.15 (0.48-2.74)
>median, n=52,696	1.00	1.46 (1.14-1.87)	1.48 (1.33-1.66)	2.02 (1.40-2.92)
BMI, kg/m^2				
<25, n= 35,583	1.00	1.45 (0.87-2.40)	1.57 (1.24-1.99)*	3.02 (1.50-6.08)*
≥25, n= 69,823	1.00	1.34 (1.07-1.69)	1.39 (1.25-1.54)*	1.54 (1.06-2.23)*
Sedentary time, h/day				
≤ median, n=56,191	1.00	1.50 (1.11-2.04)	1.47 (1.27-1.71)*	1.71 (1.07-2.75)
>median, n=49,215	1.00	1.22 (0.91-1.64)	1.37 (1.21-1.55)*	1.56 (0.96-2.54)

Logistic regression model adjusted for age, sex, ethnic background (British, other ethnic group), educational level (primary or less, secondary, and university), Townsend deprivation index (continuous), smoking status (current, former, or never), BMI (< 25.0, 25.0–29.9, ≥ 30.0 kg/m^2), sedentary time (tertiles of h/day), hours of daily sleep (<7, 7-8, >8), number of treatments/medications, except for the stratification variable.
*p for interaction <0.05, from likelihood ratio tests comparing models with and without an interaction term, defined as the cross-product of the sensory impairment and the stratification variable.

Supplementary Table 8. Odds ratios (95% confidence interval) for the association between sensory impairment and individual components of frailty syndrome in the UK Biobank. (N= 105,406)

	Sensory impairment			
	Without any impairment (n=90,598)	Visual (n=2271)	Hearing (n=12,033)	Dual n=504
Frailty phenotype				
Weight loss	1.00	0.97 (0.87-1.09)	0.96 (0.91-1.02)	1.00 (0.79-1.28)

© 2025. Yévenes-Briones H et al. Published online at http://www.aginganddisease.org/EN/10.14336/AD.2025.0342

Exhaustion	1.00	1.09 (0.96-1.25)	1.20 (1.13-1.27)	1.45 (1.14-1.84)
Low physical activity	1.00	1.24 (1.07-1.45)	1.17 (1.09-1.26)	1.30 (0.99-1.71)
Slowness	1.00	1.21 (1.03-1.42)	1.20 (1.12-1.29)	1.41 (1.07-1.85)
Weakness	1.00	1.16 (1.05-1.27)	1.15 (1.10-1.20)	1.34 (1.11-1.61)
Frail scale				
Fatigue	1.00	1.09 (0.96-1.25)	1.17 (1.10-1.25)	1.46 (1.15-1.86)
Low strength	1.00	1.11 (1.00-1.24)	1.21 (1.16-1.27)	1.14 (0.92-1.40)
Reduced aerobic capacity	1.00	1.18 (1.03-1.36)	1.18 (1.10-1.25)	1.38 (1.07-1.79)
Having several chronic illnesses	1.00	1.43 (0.66-3.09)	1.31 (0.93-1.85)	2.24 (0.95-5.27)
Unintentional weight loss	1.00	0.97 (0.87-1.09)	0.96 (0.91-1.02)	1.00 (0.79-1.28)

Logistic regression model adjusted for age, sex, ethnic background (British, other ethnic group), educational level (primary or less, secondary, and university), Townsend deprivation index (continuous), smoking status (current, former, or never), BMI (< 25.0, 25.0–29.9, ≥ 30.0 kg/m^2), sedentary time (tertiles of h/day), hours of daily sleep (<7, 7-8, >8), number of treatments/medications, and for the other components of the frailty syndrom

© 2025. Yévenes-Briones H et al. Published online at http://www.aginganddisease.org/EN/10.14336/AD.2025.0342
